# Sensorimotor function of the cervical spine in healthy volunteers

**DOI:** 10.1016/j.clinbiomech.2015.01.005

**Published:** 2015-03

**Authors:** Neil J. Artz, Michael A. Adams, Patricia Dolan

**Affiliations:** Centre for Comparative and Clinical Anatomy, University of Bristol, Bristol, UK

**Keywords:** Cervical spine, Neck muscles, Proprioception, Position sense, Movement sense, Reflex activation

## Abstract

***Background*:**

Sensorimotor mechanisms are important for controlling head motion. However, relatively little is known about sensorimotor function in the cervical spine. This study investigated how age, gender and variations in the test conditions affect measures of position sense, movement sense and reflex activation in cervical muscles.

***Methods*:**

Forty healthy volunteers (19M/21F, aged 19–59 years) participated. Position sense was assessed by determining repositioning errors in upright and flexed neck postures during tests performed in 25%, 50% and 75% cervical flexion. Movement sense was assessed by detecting thresholds to passive flexion and extension at velocities between 1 and 25°s^− 1^. Reflexes were assessed by determining the latency and amplitude of reflex activation in trapezius and sternocleidomastoid muscles. Reliability was evaluated from intraclass correlation coefficients.

***Findings*:**

Mean repositioning errors ranged from 1.5° to 2.6°, were greater in flexed than upright postures (*P* = 0.006) and in people aged over 25 years (*P* = 0.05). Time to detect head motion decreased with increasing velocity (*P* < 0.001) and was lower during flexion than extension movements (*P* = 0.002). Reflexes demonstrated shorter latency (*P* < 0.001) and greater amplitude (*P* = 0.009) in trapezius compared to sternocleidomastoid, and became slower and weaker with age. None of the measures were influenced by gender. Reliability was good for movement sense measures, but was influenced by the test conditions when assessing position sense.

***Interpretation*:**

Increased repositioning errors and slower reflexes in older subjects suggest that sensorimotor function in the cervical spine becomes impaired with age. In position sense tests, reliability was influenced by the test conditions with mid-range flexion movements, performed in standing, providing the most reliable measurements.

## Highlights

• Sensorimotor function in the cervical spine was assessed in healthy volunteers.
• Cervical spine position sense was affected by posture, movement direction and age.
• Cervical spine movement sense was influenced by velocity and direction of movement.
• Reflex activation of cervical muscles became slower and weaker with age.
• Position sense and movement sense showed no correlation with reflex responses.

## 1 Introduction

The slender and mobile cervical spine is particularly vulnerable to injury in bending (Przybyla et al., 2007), so sensorimotor processes are vital for maintaining stability and controlling movements of the head. Proprioception is an important component of sensorimotor function, providing the body with a sense of position, sense of movement, sense of force, and sense of effort. These sensations are provided by proprioceptors in muscles, ligaments tendons and skin, although muscle spindles are thought to be the receptors primarily responsible for position and movement sense (Burgess et al., 1982; Ferrell and Smith, 1988; Gandevia and Burke, 1992; Marks, 1997; Matthews, 1988). Neck muscles have a particularly high density of muscle spindles (Boyd-Clark et al., 2002; Liu et al., 2003), and these proprioceptors have anatomical connections with the vestibular and visual systems (Treleaven, 2008) suggesting that proprioceptive information is integrated with other sensory information in order to fine tune the position and movement of the head. Muscle spindles are also involved in several reflexes, including simple stretch reflexes, that are important in controlling head motion and protecting the underlying spinal tissues from injury (Keshner and Peterson, 1995; Peterson, 2004; Peterson et al., 1985; Wilson et al., 1990).

In the cervical spine, proprioceptive function has been investigated most often by evaluating joint position sense. This is generally assessed by measuring repositioning errors when subjects attempt to reproduce specific head positions, and in such tests, subjects are normally blindfolded to remove visual cues. Measurement techniques include electromagnetic tracking devices (Kristjansson et al., 2001; Swait et al., 2007), camera-based systems (Edmondston et al., 2007; Wong et al., 2006) and ultrasonography (Demaille-Wlodyka et al., 2007; Roren et al., 2009; Strimpakos et al., 2006). These methods have clinical potential because they are sensitive enough to demonstrate increased repositioning errors in people with neck pain (Kristjansson et al., 2003; Revel et al., 1991; Roren et al., 2009), and to detect improvements in response to training (Humphreys and Irgens, 2002; Jull et al., 2007). However, studies in peripheral joints and in the thoracolumbar spine suggest that measures of position sense are influenced by the test conditions with factors such as the limb (Lonn et al., 2000b) or trunk (Preuss et al., 2003) position, the range (Janwantanakul et al., 2001) and direction of movement (Carpenter et al., 1998; Swinkels and Dolan, 1998, 2000; Weiler and Awiszus, 2000) and the use of passive versus active movements (Lonn et al., 2000a; Proske and Gandevia, 2012; Silfies et al., 2007) affecting their accuracy and reliability. Such influences may be particularly important in the cervical spine where position and movement of the head in space, and relative to the trunk, are likely to have independent effects on vestibular and proprioceptive systems.

Movement sense is considered distinct from position sense and is generally evaluated by measuring thresholds to the detection of passive movement, assessed as the angular movement or the time delay between the onset and detection of motion. In peripheral joints, detection thresholds are reported to be lower during faster movements and in proximal compared to distal joints (Hall and McCloskey, 1983). In the cervical spine, there is some evidence that movement sense is influenced by speed of movement (Taylor and McCloskey, 1988) but these findings are based on a small number of subjects and only for rotational movements. Movement sense has not been assessed during flexion/extension of the cervical spine although such measures may have particular relevance when investigating people with whiplash associated disorders.

The importance of proprioception in the control of movement suggests that any impairment of position or movement sense may have adverse effects on motor control mechanisms, leading to an increased risk of injury. In the lumbar spine, delayed reflex activation of trunk muscles has been observed in people with low back pain (Hodges and Richardson, 1998; Lexell and Downham, 1991; Magnusson et al., 1996), and in healthy subjects prolonged muscle response times have been associated with an increased risk of future back injury (Cholewicki et al., 2005). These findings suggest that delayed muscle reflexes may be a cause or consequence of low back pain, but whether this is due to peripheral changes in the muscle, such as fibre atrophy, or to poor proprioceptive function is unclear. In the cervical spine, impaired proprioception has been linked with neck pain (Kristjansson et al., 2003; Revel et al., 1991; Roren et al., 2009) but the extent to which poor proprioception influences motor responses of cervical muscles remains unknown.

The aim of the present study was to assess position sense, movement sense and reflex responses of cervical muscles in healthy volunteers to determine how they are affected by age, gender and variations in the test conditions. In position sense tests, the effects of varying the test position, as well as the range and direction of movement were investigated. In movement sense tests, the effects of speed and direction of movement were evaluated. A secondary aim was to assess the reliability of these measurements and investigate correlations between them.

## 2 Methods

### 2.1 Participants

Participants aged between 18 and 60 years, with no previous history of back or neck pain requiring medical attention or time off work, were recruited by “word of mouth” and via poster advertisements around the University. Forty healthy volunteers (19 male, 21 female), mean (± STD) age 29.9 (± 10.8) years, consented to participate. All participants were subsequently screened to exclude neck pain and a history of traumatic neck injury.

### 2.2 Experimental protocol

Subjects performed a series of tests that included measures of joint position sense, movement sense, and assessment of neck muscle reflexes. Each test was performed three times and a mean value obtained. The first set of tests was carried out on a single day in a standardised order. Twenty-one participants repeated the tests on the same day, and nineteen repeated them on two separate days, at least one week apart, to enable within-day and between-day reliability to be determined. Twenty one participants also took part in a preliminary validation study of the movement sense tests. During all procedures, testing was carried out by the same examiner. The study was approved by the Research Ethics Committee of the Faculty of Medical and Veterinary Sciences at the University of Bristol.

### 2.3 Assessment of position sense

**Fig. 1:**
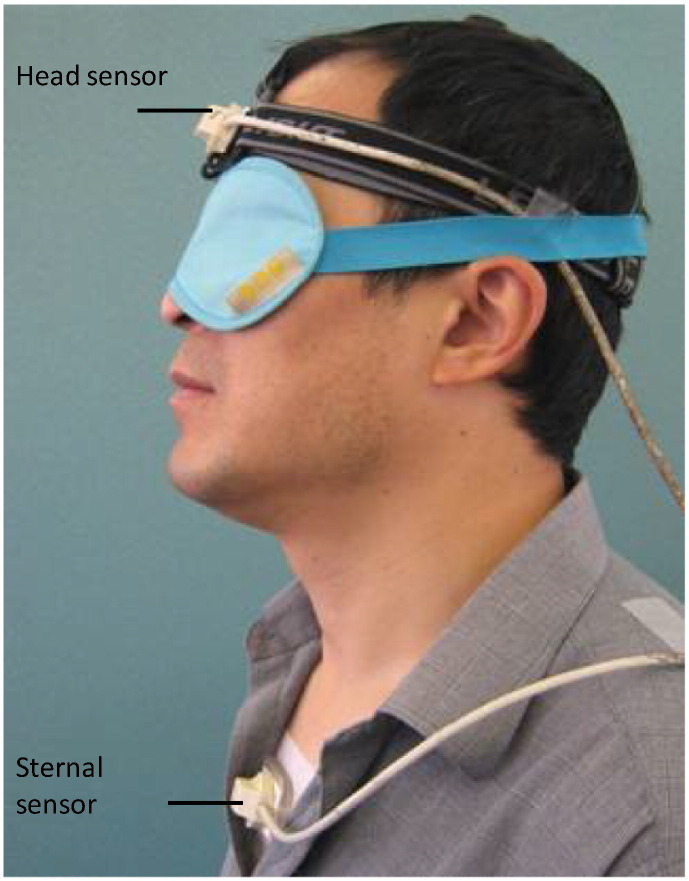
During position sense testing, movements of the head were assessed using the 3-Space Fastrak electromagnetic goniometer. One movement sensor (the Head sensor) was placed 2 cm above the glabella and another (the Sternal sensor) was placed 5 cm below the sternal notch, along the central axis of the body. (Subjects were blindfolded during testing to remove visual cues.)

Cervical spine position sense was measured using the 3-Space Fastrak (Polhemus, Inc., Colchester, VT, USA). Movement sensors, mounted on perspex base-plates, were fixed to the sternum (5 cm below the sternal notch) and the forehead (2 cm above the glabella) using Hypafix (BSN Medical, Hamburg, Germany) and double-sided tape (Fig. 1). During testing, the Fastrak source was placed within 20 cm of the subject's head, and the angular orientation of each sensor relative to the source was recorded at 60 Hz using custom-made software (Swinkels and Dolan, 1998). The angular difference between the head and sternal sensors indicated the head angle relative to the trunk. Position sense was assessed as the absolute difference in head angle when the same target posture was adopted twice in quick succession (Swinkels and Dolan, 1998).

**Fig. 2:**
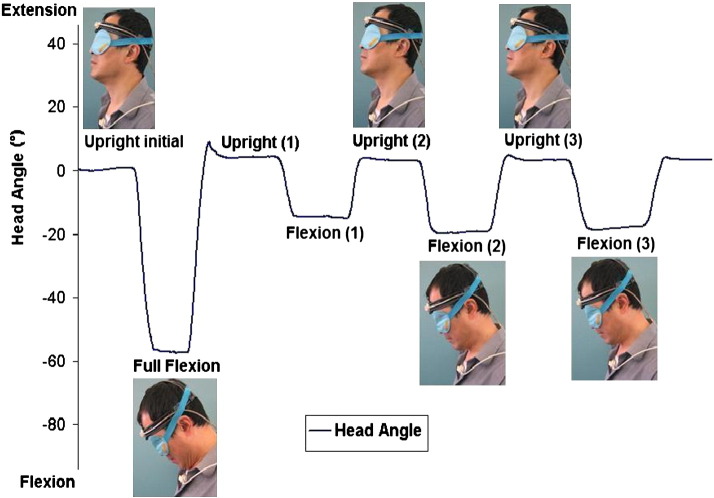
Changes in head angle during joint position sense testing. Absolute repositioning errors were calculated as the absolute angular difference between the second and third attempts to reproduce each posture i.e. Upright (2)–Upright (3) and Flexion (2)–Flexion (3), respectively. Perception of range was calculated by expressing the head angle during the first attempt to reproduce the flexed target position, Flexion (1), as a percentage of the full range of flexion between the Upright initial posture and Full Flexion.

To measure position sense in standing, subjects stood barefoot, with arms by their side. In sitting, subjects sat in a low chair with the back supported and forearms resting on the arms of the chair. The testing protocol was explained and demonstrated to each subject by the same examiner, after which subjects were blindfolded to eliminate visual cues. During each trial, subjects initially adopted the upright posture for 2 s before moving their head into full flexion and then returning to the upright posture. This indicated the full range of cervical flexion against which subjects were required to gauge subsequent target positions. They then made three attempts to adopt a given target position (25%, 50%, or 75% range of flexion) before returning to their “exact upright starting posture”, in their own time. Subjects were instructed to hold each posture for 2 s to ensure that their position is stabilised but were not given any feedback during the trials that would help them to achieve the target posture. Position sense was defined as the absolute difference in head angle between the second and third repositioning attempts within a given trial, and these “repositioning errors” were assessed for both flexed and upright postures (Fig. 2). Each subject's perception of range was evaluated from the undershoot or overshoot (expressed as % range of flexion) during the first attempt to adopt the flexed target posture. The sequence of testing (e.g. 50%, 25%, 75%) was randomised to minimise learning effects.

### 2.4 Assessment of movement sense

**Fig. 3:**
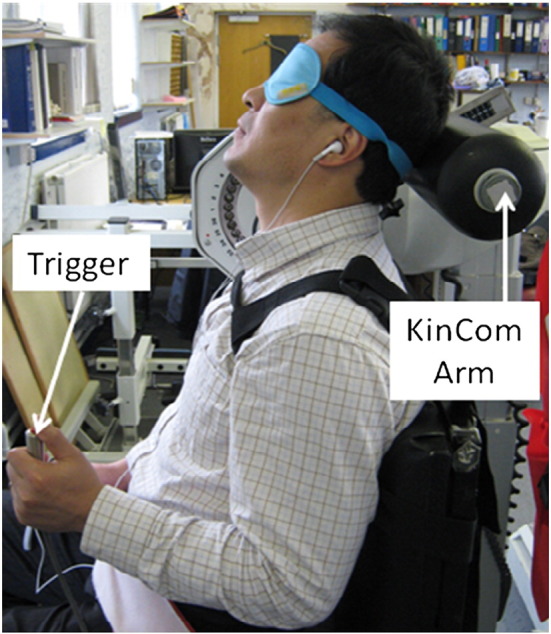
During movement sense testing, subjects were seated in a KinCom dynamometer with the neck positioned in 50% flexion or extension. The cushioned arm of the machine was positioned behind the head such that its centre of rotation was aligned with the approximate centre of the C7–T1 intervertebral disc. Subjects were secured using shoulder and waist straps, and wore a blindfold and headphones to eliminate audio-visual cues. Volunteers pressed a hand-held trigger upon detection of head movement.

**Fig. 4:**
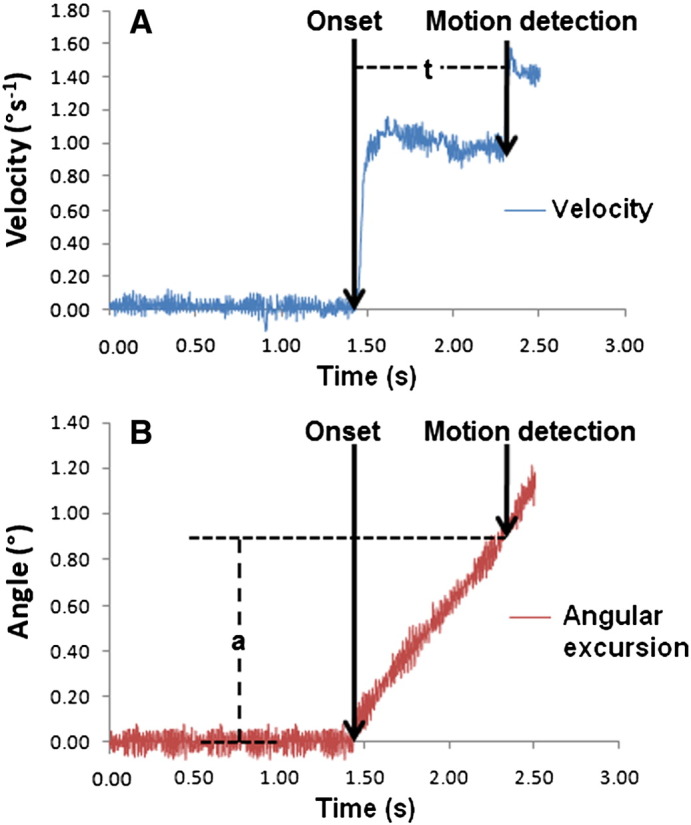
Assessment of “movement sense”. (A) Velocity trace for the KinCom at 1°s^− 1^. Time to detect head motion was determined as the time difference (ms) between the onset of motion and the point at which motion was detected (t). (B) Corresponding graph showing the angular position of the KinCom arm. The motion onset and motion detection time points were used to determine the angular movement (°) of the head (a) when motion was first detected.

Movement sense was evaluated as the ability to detect passive movement of the head over a range of velocities. Testing was carried out using a Kincom isokinetic dynamometer (Chattanooga Group Ltd, Hixson, TN, USA) which has a movable arm that can be programmed to move at different angular velocities. Subjects sat in the Kincom with the neck in 50% extension or flexion, and the head resting against the cushioned arm of the machine (Fig. 3). They were secured using shoulder and waist straps, and wore headphones and a blindfold to eliminate audiovisual cues. The Kincom was programmed to move at a predetermined velocity, and subjects were asked to press an electronic trigger as soon as they sensed that their head was moving. Angular velocity and position of the Kincom arm were recorded at 500 Hz using BioWare 3.20 software (Kistler Corp, Winterthur, Switzerland). The time taken to detect head motion, and the angular movement when head motion was detected, were recorded as measures of “movement sense” (Fig. 4).

An initial validation study was carried out in twenty-one subjects who performed tests at six velocities (1°s^− 1^, 2°s^− 1^, 3°s^− 1^, 5°s^− 1^, 10°s^− 1^ and 25°s^− 1^) in extension only. All subjects repeated the tests on the same day, and nineteen repeated the tests on a separate day at least one week later. This enabled “within-day” and “between-day” reliability to be determined across a range of velocities. In the main study, movement sense was assessed in all forty subjects during flexion and extension tests performed at two velocities (1°s^− 1^ and 10°s^− 1^). These subjects also repeated the flexion tests on the same day to enable their reliability to be determined.

### 2.5 Assessment of reflex activation

Muscle (EMG) activity was recorded using skin-surface electrodes following careful skin preparation (Dolan and Adams, 1993). Pairs of adhesive Ag/AgCl electrodes (Unomedical Ltd, Stonehouse, UK) were then applied bilaterally over the upper trapezius, 2 cm lateral to the midline at the C5/6 level, and over sternocleidomastoid, one-third of the length from the rostral to sternal attachments, with an inter-electrode distance of 2 cm (Sommerich et al., 2000). A reference electrode was placed over the sternum. Impedance between each recording electrode and the reference was checked to ensure this was below 5 kΩ. During testing, the EMG signal was recorded at 500 Hz, band pass-filtered between 8 and 500 Hz, full wave-rectified and amplified (Biodata PA400, Manchester, UK) and A-D converted for subsequent analysis (Dolan and Adams, 1993).

**Fig. 5:**
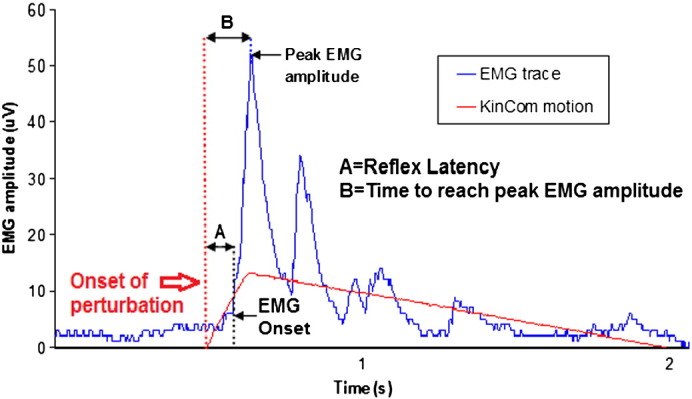
Reflex response of right trapezius muscle. Reflex latency (A) is defined as the time difference (ms) between the onset of perturbation and the EMG onset. Peak EMG amplitude is the maximum EMG response (μV) following perturbation. Time to reach peak EMG amplitude following the onset of perturbation (B) was also recorded.

Reflex responses were initiated using the Kincom dynamometer. Subjects were secured in the Kincom with their neck flexed by 50% and their forehead resting on the cushioned arm of the machine. Headphones and a blindfold were worn to remove audiovisual cues. Several seconds of baseline data were recorded after which the Kincom arm was programmed to move at 100°s^− 1^ for 150 ms in order to initiate rapid cervical flexion. The position and velocity of the Kincom arm were recorded at 500 Hz, simultaneously with the EMG data. The initiation of movement was determined from the position data, and activation of the two muscles was determined from the individual EMG traces (Fig. 5). *Reflex latency* represents the delay between perturbation and muscle activation. This was determined for each muscle by estimating the mean plus three standard deviations of the baseline activity prior to movement, and identifying the time, following perturbation, at which this value was exceeded. To be accepted as a reflex, latency had to be in the range 30–150 ms to eliminate vestibular (Ito et al., 1995) and voluntary muscle activation. *Peak EMG r*epresents the highest EMG amplitude for each muscle, and *time to reach peak EMG* was estimated as the time between the onset of perturbation and the peak EMG.

### 2.6 Statistical analysis

For each outcome measure, a separate mixed model ANOVA was used to assess the effects of various ‘within-subject’ and ‘between-subject’ factors. Outcome measures included “repositioning errors” and “actual flexion” (in position sense tests), “time to detect motion” and “angular movement threshold” (in movement sense tests), and “reflex latency”, “time to peak EMG”, and “peak EMG amplitude” (in reflex activation tests). In each ANOVA, gender and age (dichotomised into those 25 years or younger and those above 25 years) were included as ‘between-subject’ factors, and the following were included as ‘within-subject’ factors: posture (flexed or upright), range of movement (25, 50 or 75% flexion) and test position (sitting or standing) in position sense tests; direction (flexion or extension) and velocity of movement in movement sense tests; and muscle (trapezius or sternocleidomastoid) and side (right or left) in reflex activation tests. Significant interactions between factors were investigated using appropriate post-hoc comparisons. Within-day and between-day reliability was assessed using a repeated measures analysis of variance to obtain the ICC, and using the standard error of measurement. Associations between parameters were assessed using Pearson's product correlation coefficient. Significance was accepted at the 5% level.

## 3 Results

### 3.1 Position sense

Mean absolute repositioning errors are shown in Table 1. There was no significant difference between sitting and standing, and no main effect of range of movement. However, repositioning errors were greater when adopting flexed compared to upright postures (*P* = 0.006) and there was a significant interaction between posture and range of movement (*P* = 0.001). This was investigated further using repeated measures analysis of variance to compare repositioning errors separately in flexed and upright postures. These post-hoc tests showed that, with increasing target range, repositioning errors decreased in flexed postures (*P* = 0.005) and increased on returning to upright (*P* = 0.007). No differences were observed between genders but age had a marginal effect (*P* = 0.05), with older subjects (> 25 years) exhibiting greater repositioning errors. There was also a significant interaction between age and range (*P* = 0.039) which was further investigated using group t-tests to compare the effects of age within each range of movement. These post-hoc tests showed that age differences were significant only when the target range of flexion was 50% (*P* = 0.002). Consequently, average repositioning errors for each subject showed no significant correlation with age.

Closer examination of the position sense data showed that 10 subjects had a missing value across the many repeated trials which, in a repeated measures analysis, effectively reduced the sample size to 30. Repeating the ANOVA using an imputation method to take account of missing values confirmed the original findings and improved the significance levels for posture (*P* < 0.001), age (*P* = 0.044) and the interactions between posture and range (*P* < 0.001) and age and range (*P* = 0.005).

Within-day and between-day comparisons revealed no significant differences in position sense between trials. However, ICC values were highly variable ranging from − 0.81 to 0.77 (Table 1). Based on the ICC and standard error of measurement, reliability was most consistent when tests were performed in standing with a target range of 50% flexion.

The “actual flexion” achieved during testing differed from the target value (25%, 50% or 75%) with subjects consistently overshooting target postures in both sitting and standing (Table 2). The mean overshoot decreased significantly from 14% when the target was 25% flexion to 1.5% when the target was 75% flexion (*P* < 0.001). Within-day and between-day comparisons revealed no significant differences between trials, and ICC values were between 0.52 and 0.87 (Table 2).

### 3.2 Movement sense

In the validation study, the time taken to detect initial head motion decreased with increasing velocity of movement (Table 3) from 799 (SD 406) ms at 1°s^− 1^ to 299 (SD 61) ms at 25°s^− 1^ (*P* < 0.0001) whilst the angular movement threshold increased from 0.80° (SD 0.41°) at 1°s^− 1^ to 7.47° (SD 1.53°) at 25°s^− 1^ (*P* < 0.0001). Within-day and between-day comparisons revealed no significant differences between trials, and ICC values were between 0.58 and 0.88 (Table 3).

**Fig. 6:**
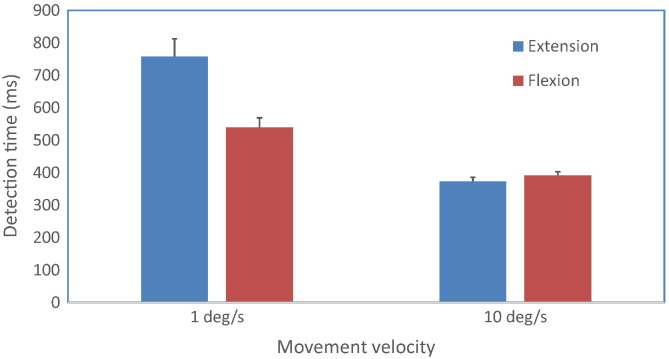
Movement sense was measured as the time taken to detect initial head motion during passive cervical extension and flexion performed at 1°s^− 1^ and 10°s^− 1^. Mean values are shown for initial tests performed by all subjects (n = 40) on the first test day. Error bars indicate the standard error of the mean.

In the main study, where movement sense was assessed at just two velocities (Fig. 6), the time taken to detect head motion was greater at 1°s^− 1^ compared to 10°s^− 1^ (*P* < 0.001) and during extension compared to flexion movements (*P* = 0.002). There was also a significant interaction between these effects (*P* < 0.001). Further analyses using matched pair t-tests to compare the effects of movement direction at each velocity showed that movement direction influenced detection times significantly at 1°s^− 1^ but not at 10°s^− 1^. No significant effects of age or gender were observed. Flexion tests repeated on the same day showed no significant differences between repeated trials, and ICC values were similar to those obtained in extension, ranging from 0.57 at 10°s^− 1^ to 0.73 at 1°s^− 1^.

### 3.3 Reflex activation

Reflex responses are summarised in Table 4. No significant differences were observed between right and left muscles. However, reflex latency (*P* < 0.001) and time to peak EMG (*P* < 0.001) were shorter and peak EMG was greater (*P* = 0.009) for trapezius compared to sternocleidomastoid. There was a trend towards longer latencies in male subjects but differences did not reach significance (*P* = 0.063). Reflex latency was greater in subjects aged over 25 years (*P* = 0.003), and was significantly correlated with age for both trapezius (R = 0.34, *P* = 0.042) and sternocleidomastoid (R = 0.50, *P* = 0.002). In contrast, peak EMG amplitude decreased with age for trapezius (*R* = 0.381, *P* = 0.018) but not sternocleidomastoid (*R* = 0.088, *P* = 0.598). Within-day and between-day comparisons showed no significant differences between trials, and respective ICC values were in the ranges 0.57–0.83 and 0.23–0.64 (Table 4).

### 3.4 Effect of position sense and movement sense on reflex activation

The latency and amplitude of reflex activation for both trapezius and sternocleidomastoid showed no significant association with measures of position sense. This was true if position sense measures were averaged over all tests, or if values were used only for the most reliable test (50% range of flexion in standing). Measures of movement sense, similarly had no effect on either the latency or amplitude of reflex activation for both muscles.

## 4 Discussion

### 4.1 Summary of findings

This study has demonstrated how variations in the test conditions, and other factors such as age and gender, affect measures of sensorimotor function in the cervical spine. In position sense tests, repositioning errors were lower when adopting upright compared to flexed postures and in people aged 25 years or younger. Reliability varied considerably across the different test conditions and was most consistent for tests performed in 50% flexion whilst standing. In movement sense tests, the time taken to detect head motion decreased at faster velocities and during flexion movements whereas the angular movement threshold decreased at slower velocities. ICC values indicated moderate to excellent reliability and were not influenced substantially by velocity or direction or movement. In reflex activation tests, muscle responses were faster and of greater amplitude for trapezius than sternocleidomastoid. Response time (reflex latency) increased with age for both muscles, whereas EMG amplitude decreased for trapezius only. Reliability was moderate to excellent for within-day trials but was reduced in between-day trials which may reflect small variations in electrode placement and skin impedance in tests performed on different days.

### 4.2 Strengths and weaknesses of the study

The repeated measures design enabled the effects of various parameters to be assessed in the same subjects who thus acted as their own controls. This minimised the confounding influence of other variables such as age and gender whilst allowing their effects within the group to be evaluated. However, repeated testing of subjects might also induce learning effects or fatigue. For this reason, adequate rest periods were allowed between tests, and as a result no significant differences were observed between repeated within-day or between-day trials suggesting learning and fatigue effects were minimal. Another potential source of error relates to muscle thixotropy, which is a property of passive muscle to change its mechanical properties in response to its recent loading history. This is related to changes in the sensitivity of muscle spindles to passive movement and may therefore affect measures of proprioception during passive testing (Proske and Gandevia, 2012). In the present study, position sense was assessed during active movements of the head and neck where thixotropic effects were expected to be small. However, movement sense was assessed using passive motion where thixotropic influences would be more evident. To minimise such effects, all tests were performed in a standardised order so that pre-conditioning of muscle would be similar for all subjects. Consequently, when movement sense tests were repeated, on the same day or on a separate day, there was no significant change in the measurements and intraclass correlation coefficients were high. These findings suggest that any thixotropic influences were at least consistent and hence were unlikely to be a confounding factor when comparing other influences on proprioception. Subjects across a wide age range were eligible for the study to enable any age effects to be identified. Nevertheless, most subjects (34 out of 40) were below the age of 40 which may explain why age effects were small and not always significant. The lower sample size obtained by dichotomising the data according to gender may have limited the ability to detect gender differences. However, such differences were generally small and inconsistent. The only exception was reflex latency which showed a trend towards greater values in men that may reflect slightly greater nerve conduction distances. Such gender effects therefore warrant further investigation in a larger scale study.

### 4.3 Relationship to other studies

In position sense tests, repositioning errors were consistent with those reported previously for the cervical spine (Christensen and Nilsson, 1999; Lee et al., 2006; Strimpakos et al., 2006) and lower than those observed in the thoracolumbar spine (Swinkels and Dolan, 1998; Swinkels and Dolan, 2000) and peripheral joints (Barrack et al., 1984; Skinner et al., 1986; Soechting, 1982). These findings suggest that position sense is highly developed in the cervical spine, reflecting the high density of muscle spindles in deep muscles of the neck (Amonoo-Kuofi, 1982; Boyd-Clark et al., 2002; Liu et al., 2003). The lower repositioning errors in upright compared to flexed postures confirm earlier findings in the thoracolumbar spine (Swinkels and Dolan, 1998) and may suggest a greater contribution to position sense from the vestibular system when adopting upright postures (Swinkels and Dolan, 1998). Interestingly, subjects in the present study showed a reduced ability to reproduce upright postures but an improved ability to reproduce the flexed target when the range of flexion during testing was increased. Previous work in the thoracic and lumbar spine suggests that position sense is independent of the range of movement across intermediate ranges (Swinkels and Dolan, 2000) although there is some evidence of improved accuracy during larger ranges of trunk movement (Allison and Fukushima, 2003). A possible explanation is that afferent feedback from ligamentous mechanoreceptors increases as tension in posterior ligaments increases, contributing to greater repositioning accuracy in more flexed postures. Reduced accuracy on returning to the upright posture may reflect thixotropic effects in muscle spindles, which have a “load memory” and become relatively less sensitive after previous stretching of the muscle (Ge and Pickar, 2008). The particularly high density of muscle spindles in cervical compared to lumbar muscles (Peck et al., 1984) may contribute to this effect in the cervical spine.

The lack of any gender effects on position sense is consistent with previous findings in the cervical (Demaille-Wlodyka et al., 2007; Heikkila et al., 1996; Sterling et al., 2003) and lumbar (Feipel et al., 2003) spine. However, conflicting results have been reported regarding age effects. In the current study, subjects over 25 years old exhibited increased repositioning errors, but only for tests performed in the mid-range of movement. Consequently, mean repositioning errors showed no significant correlation with age. Previous studies have similarly found no correlation between cervical spine position sense and age in healthy controls (Heikkila and Wenngren, 1998; Rix and Bagust, 2001). However, age-related declines in position sense have been reported in the knee (Hurley et al., 1998; Skinner et al., 1984), lumbar spine (Goldberg et al., 2005) and cervical spine (Lansade et al., 2009; Vuillerme et al., 2008) in people aged over 60 years. Recent evidence suggests that age-related declines in muscle function, including sensorimotor performance, do not occur until the sixth decade (Deschenes, 2004; Vandervoort, 2002) which could explain the conflicting results in the literature.

Reliability of position sense measurements was highly variable, as reported previously (Strimpakos et al., 2006) and this raises questions regarding the clinical usefulness of such measures. ICC values can be low because within-subject variability is high (indicating poor reliability) or because between-subject variability is low. In the latter case, the standard error of measurement can provide additional information concerning reliability. In the present study, standing tests with a target range of 50% neck flexion achieved the highest ICC and lowest SEM values (Table 1) suggesting that this format would be most suitable for future studies. Tests performed over this range also demonstrated a significant dependence upon age, which might be linked to the greater reliability of the measurements. Differences between target and actual “% flexion” were reduced in more flexed postures, where ICC values also improved. Accuracy in achieving a pre-set target flexion angle could therefore be used alongside absolute repositioning error as a reliable measure of position sense in the cervical spine.

Movement sense in the cervical spine proved to be velocity-dependent with faster detection times but greater angular thresholds to movement detection at higher velocities. In the validation study, the fall in detection time was significant, even when comparing the two fastest velocities, suggesting that the measures were not simply reflecting the reaction time of subjects in pressing the trigger. The neck muscles are well-endowed with muscle spindles (Amonoo-Kuofi, 1982; Peck et al., 1984) which contain tonic fibres that respond to slow or sustained stretch, and phasic fibres which provide the dynamic response that contributes to the stretch reflex. Spindles respond more rapidly to greater rates of stretch (Proske et al., 2000) which may explain why shorter detection times were observed at the faster velocities. An earlier study found that angular thresholds to movement detection were greater at slower velocities, although the range of velocities examined (between 0.1 and 5.7°s^− 1^) were somewhat lower than in the present study (Taylor and McCloskey, 1988). At very slow rates of movement, it is possible that only tonic fibres with slower response times would be activated and this could explain the conflicting findings. The faster detection times observed during flexion may reflect the high density of spindles in muscles of the sub-occipital triangle (Kulkarni et al., 2001; Peck et al., 1984). These small muscles are thought to act as sensors of craniovertebral motion, and because of their location they will be subjected to relatively high levels of stretch during flexion which may contribute to increased sensitivity to flexion movements.

The effects of age and gender on movement sense have not been investigated previously in the cervical spine, although the current study found that neither had any influence on detection times or angular thresholds to movement detection. As mentioned previously, most age-related changes in muscle function tend to occur in people aged over 50, and this may have contributed to the lack of effect in the current cohort where subjects were mostly under 40 years of age.

ICC values for movement sense in the cervical spine (Table 3) indicate generally good levels of reliability in the present study, consistent with previous findings in the lumbar spine (Silfies et al., 2007).

Reflex responses were approximately 30% faster and 20% greater for trapezius than sternocleidomastoid. The most rapid reflexes are monosynaptic stretch reflexes initiated by muscle spindles, which typically have a latency of 30–50 ms (Wilder et al., 1996). In this study, the reflex latency of 69 ms for trapezius indicates a poly-synaptic M_2_ reflex (Wilder et al., 1996). These findings are consistent with those of earlier studies which reported reflex latencies of 50–80 ms in cervical (Ito et al., 1997; Weerdesteyn et al., 2008) and lumbar muscles (Moseley et al., 2003; Radebold et al., 2001; Sanchez-Zuriaga et al., 2010). Sternocleidomastoid latency in the current study (100 ms) also indicates a poly-synaptic M_2_ response rather than a voluntary response since voluntary activation generally occurs with a time delay of 150 ms or more, even in elite athletes (Tonnessen et al., 2013). These findings suggest that, following some perturbation in flexion, trapezius acts primarily to limit cervical flexion whilst sternocleidomastoid is activated later to stabilise the cervical spine following the motion.

The increased latency and reduced amplitude of reflex activation observed with age is consistent with findings in the back muscles where reflex latencies of lumbar multifidus and erector spinae muscles were reported to be 12% longer in older compared to younger adults (Hwang et al., 2008). These changes in reflex activation may reflect altered motor output rather than impaired sensory input because age had only a limited effect on position sense and movement sense in the present study. The lack of correlation between measures of reflex activation and measures of position and movement sense lends some support to this suggestion. Age-related changes in muscle such as atrophy and loss of type II muscle fibres, and a slowing of nerve conduction velocity, are well documented (Vandervoort, 2002), and age-related changes in the size and number of type II muscle fibres have been observed in the back muscles (Mannion et al., 2000). Little is known about age-related changes in trapezius and sternocleidomastoid, but similar changes to those in the back muscles may explain why older subjects in the current study had slower and weaker reflexes.

Reflex parameters showed moderate to good reliability, consistent with values reported previously for spinal muscles (Sanchez-Zuriaga et al., 2010). The current findings suggest that reflex latency of trapezius following a flexion perturbation was the most reliable measure of reflex activation in the neck muscles.

## 5 Conclusions

These results have demonstrated how variations in the test conditions affect the accuracy and reliability of position sense and movement sense measures in the cervical spine, highlighting the importance of standardizing the test conditions in future studies. Age had a marginal effect on position sense but a marked effect on muscle reflexes which became slower and weaker in older people. The lack of correlation between measures of reflex activation and measures of position sense and movement sense suggests that age-related changes in muscle reflexes are caused by peripheral changes in the muscle rather than impaired sensory input.

## Conflict of Interest

None of the authors have any conflict of interest.

## Figures and Tables

**Table 1:** Mean (SD) values of absolute repositioning errors for the initial set of tests performed by all subjects (n = 40) on the first test day. Intraclass correlation coefficient (ICC) and standard error of measurement (SEM) values are based on repeated within-day (n = 21) and between-day (n = 19) measurements.

		Target range of flexion					
		25%		50%		75%	
		*Upright*	*Flexed*	*Upright*	*Flexed*	*Upright*	*Flexed*
Standing	*Repositioning error* (*°*)	1.54	2.61	1.81	2.15	2.02	1.99
	*Repositioning error* (*SD*)	(0.73)	(1.21)	(0.96)	(1.33)	(0.88)	(1.02)
	*Within-day: ICC*	0.64	0.66	0.52	0.65	− 0.81	0.07
	*Within-day: SEM* (*°*)	0.71	1.28	1.06	1.05	1.48	1.38
	*Between-day: ICC*	− 0.48	0.36	0.61	0.58	0.06	0.77
	*Between-day: SEM* (*°*)	0.98	0.90	0.72	0.78	0.99	0.80
Sitting	*Repositioning error* (*°*)	1.61	2.36	1.91	2.38	2.25	1.79
	*Repositioning error* (*SD*)	(0.97)	(1.23)	(1.13)	(1.39)	(1.42)	(0.92)
	*Within-day: ICC*	0.68	− 0.11	− 0.06	0.64	0.17	0.49
	*Within-day: SEM* (*°*)	0.91	1.48	1.31	1.32	1.44	0.97
	*Between-day: ICC*	0.27	0.58	0.51	0.09	0.49	0.11
	*Between-day: SEM* (*°*)	0.88	0.82	1.19	1.22	1.04	0.87

**Table 2:** Mean (SD) values of “actual flexion” during position sense tests shown as a percentage of the full range of cervical flexion. Values are based on the first attempt to achieve the target position during the initial set of tests on the first test day (n = 40). Intraclass correlation coefficient (ICC) and standard error of measurement (SEM) values are based on repeated within-day (n = 21) and between-day (n = 19) measurements.

		Target range of flexion		
		*25%*	*50%*	*75%*
Standing	*Actual flexion* (*%*)	39 (10)	60 (10)	77 (11)
	*Within-day: ICC*	0.59	0.86	0.80
	*Within-day: SEM* (*%*)	8	6	6
	*Between-day: ICC*	0.78	0.82	0.85
	*Between-day: SEM* (*%*)	6	6	5
Sitting	*Actual flexion* (*%*)	40 (9)	60 (10)	76 (10)
	*Within-day: ICC*	0.52	0.78	0.83
	*Within-day: SEM* (*%*)	7	7	6
	*Between-day: ICC*	0.80	0.86	0.87
	*Between-day: SEM* (*%*)	6	6	5

**Table 3:** Mean (SD) values of the time taken to detect head motion and the associated angular movement threshold during movement sense tests. Values are shown for the initial set of tests performed by all subjects in the validation study (n = 21) on the first test day. Intraclass correlation coefficient (ICC) and standard error of measurement (SEM) values are based on repeated within-day (n = 21) and between-day (n = 19) measurements. Angular movement thresholds are a product of the velocity of movement and the detection time so their ICC values are equivalent to those shown for detection times.

	Extension velocity (deg/s)					
	1	2	3	5	10	25
*Time to detect head motion* (*ms*)*: mean* (*SD*)	799 (406)	496 (190)	450 (97)	439 (95)	376 (98)	299 (61)
*Within day: ICC* *Within day:* SEM (ms)	0.88 195	0.79 122	0.58 89	0.66 77	0.67 77	0.80 45
*Between day: ICC* *Between day:* SEM (ms)	0.85 217	0.86 134	0.63 126	0.81 71	0.79 71	0.84 45
*Angular movement threshold* (*°*)*: mean* (*SD*)	0.80 (0.41)	0.99 (0.38)	1.34 (0.29)	2.19 (0.48)	3.7 (0.98)	7.47 (1.53)
*Within day:* SEM (ms)	0.19	0.12	0.09	0.08	0.08	0.04
*Between day:* SEM (ms)	0.21	0.13	0.13	0.10	0.07	0.04

**Table 4:** Mean (SD) values for reflex latency, time to reach peak EMG, and peak EMG amplitude are shown for initial tests performed by all subjects (n = 40) on the first test day. Intraclass correlation coefficient (ICC) and standard error of measurement (SEM) values are based on repeated within-day (n = 21) and between-day (n = 19) measurements. Data are shown for upper trapezius (TRAP) and sternocleidomastoid (SCM) muscles in response to rapid flexion at 100°s^− 1^, and values are averaged for right and left muscles. *Significant differences between trapezius and sternocleidomastoid (*P* < 0.001).

	Reflex latency (ms)		Time to peak EMG (ms)		Peak EMG amplitude (μV)	
	*TRAP*	*SCM*	*TRAP*	*SCM*	*TRAP*	*SCM*
*Mean* (*SD*)	69 (21)*	100 (21)	133 (36)*	176 (31)	26 (11)*	20 (12)
*Within-day: ICC*	0.83	0.57	0.79	0.59	0.64	0.83
*Within-day: SEM*	18	21	27	30	9	8
*Between-day: ICC*	0.62	0.34	0.49	0.41	0.23	0.64
*Between-day: SEM*	19	16	31	30	8	8
